# Cold Affusion in Hooping-cough

**Published:** 1841-04

**Authors:** 


*Cold Affusion in Hooping-cough.*—[The following is an abstract of Dr. Hannay's paper, on the washing of the chest with cold water in pertussis, read at the late meeting of the British Association.]

To this enlightened audience it is quite unnecessary to delineate the phenomena of the disease, or describe the various theories entertained respecting its nature. One practical fact I would bring strongly before the section, that is, the susceptibility to the impression of cold on the economy, as shown in the frequent supervention of bronchitis, pleurisy and peripneumonia, and other diseases, of which cold is the most frequent and certain exciting cause. Now, for this high sensibility to the impression of cold, no remedial measure is so likely to prove efficacious as cold washing of the chest. The efficacy of cold washing in bronchitis, of a chronic character, has been long acknowledged: and whatever may be the other pathological states (and others than mere bronchitis there must be) existing in pertussis, this one is necessarily present, therefore cold washing *must* prove serviceable. As an invigorating or tonic power, its virtues, too, were useful in pertussis, particularly in the advance of the disease, when various forms, degrees, and extents of gastro-intestinal irritation exerted and produced impaired power of the digestive organs.

The efficacy of cold ablution in many spasmodic diseases, and amongst which there were reasons for placing pertussis, led to the belief that it proved beneficial as an antispasmodic in the cure of this disease; and the author of the memoir averred that he had derived from cold washing, on this principle, much benefit in laryngismus stridulus, decidedly a spasmodic affection. It also, when done as he directs, proves powerfully rubefacient—a class of agents which, time out of mind, have been popular in the treatment of pertussis. But it surpasses the ordinary rubefacients in fulfilling other and important indications, which none of the ordinary remedies of this class effect. It is antiphlogistic, and proves also invigorating, restoring digestion, and by its antispasmodic powers allaying the spasmodic cough. After relating some cases to show its power, the author described his mode of washing, and entreated particular attention to his method, as the only way of securing his results.

It should be done in a warm apartment. The coldest water, to which a little vinegar, alcohol, or eau de Cologne, should be added, is to be used, enveloping the hand in a towel. It is to be dipped into the epithem, and the washing speedily, very speedily, performed over the whole chest, which is to be dried with a towel, previously warmed, so that a red glow or reaction may be produced. This is to be repeated twice at least, nay, three or four times each day.

In this way the author states he has cured many cases in a few days; and in all has much shortened the duration of the disease. He is not deterred from using it *in the way he describes*, even by the existence of febrile bronchitis; and, though not very decided on this point, would not hesitate to employ it, even in cases complicated with peripneumonia. He has used it at all seasons, and in all cases; and not only with perfect impunity, but with advantage. Such is a brief outline of the author's views, which he pledged himself to publish and illustrate with cases. One or two, of a striking character, of these cases were laid before the section. One vast superiority of the remedy appeared to the author to be the possibility of getting it applied; whereas, remedies cannot easily be forced on children ill of pertussis.—*Lancet. Boston Med. and Sur. Jour., March* 3.

